# Working With the Predictable Life of Patients: The Importance of “Mentalizing Interoception” to Meaningful Change in Psychotherapy

**DOI:** 10.3389/fpsyg.2019.02173

**Published:** 2019-09-26

**Authors:** Patrice Duquette, Vivien Ainley

**Affiliations:** ^1^Private Practice, Birmingham, MI, United States; ^2^Lab of Action and Body, Royal Holloway, University of London, Egham, United Kingdom

**Keywords:** interoception, mentalization of interoception, emotion, predictive processing, free energy principle, psychotherapy, psychotherapeutic change

## Abstract

To understand our patients and optimize their treatment, psychotherapists of all theoretical orientations may benefit from considering current scientific evidence alongside psychodynamic constructs. There is recent neuroscientific evidence that subjective awareness, feelings and emotions depend upon “interoception,” defined as the neural signaling to the brain from all tissues of the body. Interoception is the obvious basis of homeostasis (in the brainstem) but some interoceptive signals rise above this level and contribute to inferential processes that substantiate intrapersonal and interpersonal experience. The focus of this paper is on the essential role that their “interoception” plays in our patients’ emotional experience and subjective awareness, and how the process referred to as “mentalizing interoception” may be harnessed in therapy. This can best be understood in terms of “predictive processing,” which describes how subjective states, and particularly emotion, are inferred from sensory inputs – both interoceptive and exteroceptive. Predictive processing assumes that the brain infers (probabilistically) the likely cause of sensation experienced through the sense organs, by testing this sensory data against its innate and learned “priors.” This implies that any effort at changing heavily over-learned prior beliefs will require action upon the system that has generated that set of prior beliefs. This involves, quite literally, acting upon the world to alter inferential processes, or in the case of interoceptive priors, acting on the patient’s body to alter habitual autonomic nervous system (ANS) reflexes. Focused attention to bodily sensations/reactions, in the safety of the therapeutic relationship, provides a route to “mentalizing interoception,” by means of the bodily cues that may be the only conscious element of deeply hidden priors and thus the clearest way to access them. This can: update patients’ characteristic, dysfunctional responses to emotion and feelings; increase emotional insight; decrease cognitive distortions; and engender a more acute awareness of the present moment. These important ideas are outlined below from the perspective of psychodynamic psychotherapeutic practice, in order to discuss how relevant information from neuroscientific theory and current research can best be applied in clinical treatment. A clinical case will be presented to illustrate how this argument or treatment relates directly to clinical practice.

## Introduction

Patients undergoing psychotherapeutic work, in any theoretical orientation, often struggle to identify and verbalize their emotional experiences and to explain their subjective views of the world, in the face of the confusing messages arising from within their physical bodies, which are accompanied by their persistent, strongly-held beliefs about themselves and the world. A common experience brings individuals to our office:

“*It seems that people spend most of their time with the delusion that they have an accurate representation of the world. Actually, evidence suggests that we are all rather poor at letting our sensory experience update our beliefs, and that we are susceptible to prior beliefs and social constraints that greatly limit our ability to deal with evidence rationally. For most of us, this may be manifest*…. *as vulnerability to biases as we try to model the world*” (Fletcher and Frith, 2009, p. 52).

At any given moment, physical sensations can lead the way into a distorted view of ourselves and of reality. Emotions, especially of fear, can dominate subjective experience; biasing our assessment of what is truly emotive in our bodies and what actual meaning this may have in the current moment. Prior beliefs or expectations stimulate reactive processes, quickly defining subjective experience, allowing little room for any testing of these potentially distorted beliefs against reality. For example: if the patient’s heart races, they tend to believe they must be scared and that they face real danger; if they have tears in their eyes they claim this is the result of some hurt and another person is responsible. Whether they are trying to: describe emotional states; parse out elements of experience; or ascertain the reality of events and interactions versus those they imagine, patients often struggle to constrain the habitual influence that their body has on these processes.

Such rapid knee jerk reactions to stimuli are learned in early infancy, where all sensation feels forever and is mostly a surprise. We are “*born too early*” (Bar-Levav, 1988), such that the processes of bodily experiences that shape emotional processes are initiated before the world can be comprehended or tested against reality. Crucially, as the body changes and adapts, the brain is simultaneously establishing expectations about relationships and the environment, within and without. These early embodied patterns persist throughout life, strongly influencing how individuals understand, behave and experience the world, intra- and inter-subjectively. What our patients know of themselves and the world is “in their bones” – acquired in childhood from experience comprised of motoric, humoral, neural, sensory and autonomic responses to salient stimuli.

Neuroscience has recently increased our knowledge of the vital processes that send neural information from the body to the brain – regulating life processes at basic levels, while also modulating emotional experience and subjective awareness at the most complex mental levels. This process is termed “interoception,” which refers to the constant flow of signals passing between the body and the brain that are the “*foundation for the sequential integration of your homeostatic condition with your sensory environment, with your motivational condition, and with your social condition*” (Craig, 2008, p. 281).^1^ At an elemental level, interoception instantiates physiologic homeostatic regulation of the body and is part of the neural infrastructure that determines emotional experience and subjective awareness, ultimately influencing cognition and behavior. When theorists claim that the processing of interoceptive information from the body underpins the processes of emotion generation, feelings and affect, they are talking literally about gut feelings.

It is now recognized that interoceptive signals, combined with information from other exteroceptive sensory modalities (like vision and touch) are integrated with top-down learned expectations in the brain, thus contributing not only to homeostasis but crucially to emotion – thus ultimately influencing cognition and behavior (Critchley and Harrison, 2013). The rubric of “predictive processing” is a valuable model within which to consider current research and possible therapy. The basic premise in predictive processing is that humans do not have direct access to the truth about our internal and external environment but that our brains must make inferences about these, on the basis of the sensory evidence that we have (Friston, 2010, 2013; Pezzulo, 2014; Clark, 2016). This approach stresses that the brain’s task is to minimize the difference between the actual incoming sensory data and what the brain expects or infers (i.e., “predicts” from experience) as the most likely cause of whatever the sensory organs are currently registering (Edwards et al., 2012; Barrett et al., 2016; Seth and Friston, 2016; Critchley and Garfinkel, 2017).

At the leading edge of research, the over-arching principle of “free energy” accounts for how inferential processes support humans’ inherent drive toward homeostasis and self-organization, by minimizing uncertainty, which is defined as the difference between the actual states of the body and the states the brain infers are optimal for its Darwinian success (Friston, 2010). The insights that these new ideas offer about how individuals experience themselves, other people and the world in general adds meaningful dimensions to therapeutic practice that were unavailable even two decades ago.

“Interoceptive inference” is the specific aspect of predictive processing which refers to how we interpret internal sensations (Gu and FitzGerald, 2014; Ondobaka et al., 2017; Owens et al., 2018; Allen et al., 2019). Much of interoceptive signaling is unconscious, or at the very borders of awareness (Adam, 2010), involving the pre-reflective, sub-personal assimilation of interoceptive bodily cues. However, implicit contextualization of autonomic reflexes, and reactions to emotionally salient cues, occurs in the body all the time. These processes are generally not available to awareness but they nevertheless have powerful impact on emotions and feelings states and also on behavior – potentially leading to persistent difficulty in our patient’s lives.

In this paper, we propose that the “mentalization” of interoceptive sensations is elemental in making these pre-reflective processes available for self-reflection. The verbalization or expression of what the patient finds on self-reflection is the important starting point between patient and therapist – providing language for feeling states and bringing emotion to the level of subjective experience – which is an important goal for any psychological school of thought.

We refer to this process as “mentalizing interoception.” While the term “mentalizing” is commonly used to denote inferring or understanding the mental states of others it also refers crucially to mental states of the self (e.g., Fonagy and Target, 2006). We use the term “mentalizing” here with the ultimate goal that the patient will have an “*intentional mental state*” (Fonagy and Target, 2006; Allen et al., 2008; Bateman and Fonagy, 2012), our usage differs in that we are assuming that the term “mentalization” specifically includes inferring the imagined causes and implications of sensation that impact on the individual (Besharati et al., 2015; Fotopoulou, 2015; Fotopoulou and Tsakiris, 2017).

Following Fotopoulou and Tsakiris (2017), we assume that the backdrop to mentalizing interoception (which they also call “*embodied mentalization*”) is the “*on-going, dynamic process of maintaining and updating generative models of the likely cause of sensory data from inside the body itself and the external world*” (Fotopoulou and Tsakiris, 2017). However, while Fotopoulou and Tsakiris (2017) are principally concerned with how the infant’s development of the experience of the self requires mentalization of interoception, we focus here on the mentalization of interoception as the ongoing process of intentional, self-reflective evaluation of interoceptive sensation that can occur in the immediate present for the adult patient.

The crux of our argument is the proposal that attention to interoceptive sensation can be harnessed in therapy to support change, given the essential role that interoception plays in our patients’ emotional experience and their subjective awareness. Specifically, our purpose is to show how the patients’ mentalization of interoception can lead to the generation of newly imagined possibilities regarding current interoceptive sensations, thus bringing ongoing interoceptive inferences into awareness at a self-reflective level. Within relational interactions with the therapist, the patient can then test their sub-optimal but habitual prior beliefs, about themselves and the world (and their consequent emotions and behaviors) and create alternative, more flexible opportunities for experience and action.

Within any clinical approach, a great deal can be gained if psychotherapists comprehend: the full significance of interoception and physiological regulatory processes for subjective experience; the power of inferential processes in how we all make meaning of our sensory world; and our reliance on habitual reactions as we try to limit the uncertainty that is inherent in human experience. Understanding processes that are constantly active but often only evidenced in bodily signatures can inform and anchor the therapeutic interaction, as the patient engages in the task of generating new hypotheses (and corresponding language) and thereby creating alternative perspectives to loosen the bounds of long-held, over-determined ways of seeing, relating, and behaving in their world. We will bring to the fore current theory and research that is most relevant for practicing clinicians and will consider how these ideas can add to their practice.

Firstly, we briefly describe the neurobiology of interoception and its place in homeostatic and allostatic regulation and thus in physiologic stability, together with an outline of the embodied (interoceptive) nature of emotion and subjective experience. All-important to our argument is the manner in which an individual’s model of the world is shaped by the interaction of their interoceptive signals with higher order inferences, in the form of early (unconscious) “prior beliefs” that may be partly innate but are also learned. Crucially, these inferential processes depend on the minimization of uncertainty through prediction. Such prior beliefs have great potential for distortion and we suggest means by which therapists can identify the state of the patient’s interoceptive inferential processes and hence gain insight into their health and psychopathology. Possible interventions are suggested whereby clinicians may encourage meaningful introspection and emotional openness in patients, while relationally supporting their efforts to alter long-held perspectives about their embodied experience and its effect on their view of themselves and the world.

It is hoped that applying the lessons from predictive processing and free energy to the therapeutic process will encourage conversations across theoretical lines, while increasing collaboration between researchers in neuroscience and psychology.

## Interoception and Physiologic Regulation

We learn in childhood that we have five senses with which to experience the world (and ourselves within it) but this classic account neglects that we also perceive the world through sensations generated from within our own body, where every cell contributes to our experience, i.e., through interoception. Memories and learned associations contribute to this process (Craig, 2002, 2008; Critchley and Harrison, 2013; Ceunen et al., 2016; Khalsa and Lapidus, 2016). The interoceptive pathway originates in cells in all types of tissues of the body – including muscles, joints, teeth, skin and all the viscera (Craig, 2002) and these interoceptive signals flow to the brain through designated neural fibers (Critchley and Harrison, 2013) (see Box 1).

**BOX 1. Neuroanatomy of interoception.**

The neural pathway of interoception originates in the various tissues of the body in small diameter fibers (A and C-delta type) which transmit neural signals regarding pain, temperature, blood osmolality, and metabolic needs. These include nociceptors, thermoreceptors, osmoreceptors, and metaboreceptors. The afferent fibers collecting neuronal signals from receptors within the body transmit neural signals to lamina I – a layer of tissue that extends up through the spinal cord to the brain (“Afferent” = from the body to the brain, “efferent” = from the brain to the body). Within the brainstem, fibers carrying interoceptive information interact extensively with both branches of the autonomic nervous system (ANS), allowing a nearly instantaneous response to interoceptive neural information and thus heightening homeostatic autonomic control (Craig, 2008). Spreading into the brain, small diameter fibers project to multiple neuroanatomic areas including: nuclei within the periaqueductal gray (PAG); the parabrachial nucleus (PBN); the nucleus of the solitary tract (NTS); the thalamus (notably the Ventromedial Nucleus); and insular cortex (IC).

**FIGURE 1:**
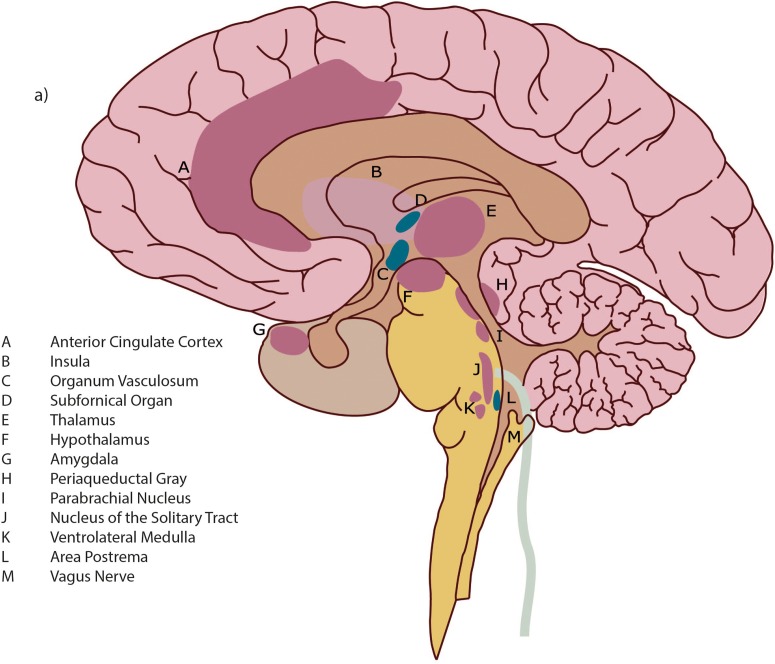
Detail of brain architecture. Reprinted with permission from Quadt et al. (2018) Annals of the New York Academy of Sciences.

The IC is a cortical area that is deeply folded and set within the large sulcus, or groove, of between the frontal and temporal lobes (see Figure 1). Neuronal signals progress through the different sections which have different cellular architecture and functional purposes; these are the posterior, middle and anterior insular cortices. For greater detail on the functional purposes of the different insular cortical sections see Box 2.

The insula is an important brain hub with wide interconnections. As well as integrating interoceptive signals, the insula receives direct input from the exteroceptive sensory cortex (for sensation from external organs, e.g., hearing, vision etc.) (Northoff, 2016). There are bidirectional connections from the insula to several areas, the prefrontal cortex, parietal and temporal cortex, basal ganglia, with the connections to the anterior cingulate cortex (ACC) heavily studied and elucidated.

The ACC plays an important complementary role to the anterior insula cortex (AIC). Most researchers agree that the AIC and ACC serve as interdependent arms of a coordinated system, which has been described as “limbic sensory” (AIC) and “limbic motor” (ACC) cortices (Craig, 2009b). While the anterior insula is assumed to underpin all feelings and awareness, the anterior cingulate is related more to motivation and behavior (Medford and Critchley, 2010; Craig, 2011). The coordinated function of the AIC and the ACC creates integrated awareness of our cognitive, affective and physical state, which then serves as the basis for the selection of, and preparation for, our responses to internal and external events (Medford and Critchley, 2010).

Interoception is functionally fundamental to homeostasis, which is largely determined unconsciously, countering the inherent instability of the organism and maintaining internal physiologic order amidst the stressor of the ever-changing external environment (Cannon, 1932). Importantly, interoceptive signals produce sensations which are experienced as pleasant or unpleasant, creating motivation within the individual (consciously or not) to move toward or away from the sensation (Craig, 2008, 2010; Duquette, 2017). The effect of this is that the flow of interoceptive signals motivates the behavior that is necessary to maintain homeostatic equilibrium – hence the essential role of interoception in motivated action (Craig, 2002, 2009b; Strigo and Craig, 2016; Critchley and Garfinkel, 2017). While homeostasis has previously been characterized as primarily reactive control of physiologic states, the concept of “allostatic” regulation is more relevant over any extended time period, as this invokes prospective control, to avoid problematic deviations from homeostatic set-points (Sterling, 2012, 2014; Stephan et al., 2016; Petzschner et al., 2017).

Allostasis, defined as “*stability through change*,” (Sterling, 2014, p. 1192) utilizes interoceptive information to implement control of bodily states in order to anticipate energy demands in advance of perturbations which would otherwise be dangerous (Sterling, 2012, 2014; Barrett et al., 2016; Stephan et al., 2016). Allostatic mechanisms are implemented either within the internal bodily milieu, by ANS reaction, or brought about by the organism’s motor behavior enacted within the environment. It is crucial for the brain to infer (i.e., to anticipate) allostatic needs, in order to engage in effective action selection and thus avoid harm (Stephan et al., 2016). For our purposes it is important to note that perturbations to allostasis can occur not only from the physical but also from the social environment of the individual.

## Interoception, Emotion, and Subjective Experience

Current theories generally accept that emotion is embodied (Frijda, 1986, 2007; Damasio, 1994, 2010; Panskepp, 1998; Barrett et al., 2004; Wiens, 2005) and that interoceptive sensation is directly related to the functional purpose of emotion. Emotion can thus be said to be: motivation which maps the rewarding/punishing aspects of stimuli to the action system for approach/withdrawal (Rolls, 1999); necessary to compute what a stimulus means to the individual (LeDoux, 2002); and “*change in action readiness to maintain or change one’s relationship to an object or event*” (Frijda, 2007, p. 158). It is interoceptive sensation itself that comprises the signal, from the body to the brain, of motivational state, with ANS sequelae as an ultimate effector arm of this processing (Craig, 2015).

What is involved in the subjective experience of embodied emotional states continues to be a subject of intense theoretical debate and this has high relevance for therapy. Two prominent early theories – the James-Lange theory (James, 1890) and that of Schachter and Singer (1962) – have claimed that we experience emotion as a result of cognitions that evaluate the changes that we perceive in the state of our body. Notably, Schachter and Singer (1962) argued that emotion involves the top-down contextualization of bodily experience by past expectations or current environment. “Bodily experience” in both these theories is what is now referred to as interoception.

In support of this, there is now substantial neuroscientific evidence that the subjective experience of emotion is generated from the integration of interoceptive signals with other sensory input, as well as top-down influences (Paulus and Stein, 2006; Harrison et al., 2010; Zaki et al., 2012; Gu et al., 2013a; Adolfi et al., 2017). Moreover, the *subjective awareness* of emotion is similarly based on interoception (Cameron, 2001; Critchley et al., 2004; Craig, 2009b; Singer et al., 2009; Damasio, 2010; Paulus and Stein, 2010; Berntson et al., 2011; Seth et al., 2011; Critchley and Nagai, 2012; Gu et al., 2012, 2013a; Herbert and Pollatos, 2012; Jones et al., 2015) (see Box 2).

**BOX 2. The subjective experience of emotion.**

Drawing on a wide range of research, Craig concludes that the convergent evidence “*implies directly that the AIC supports awareness of the immediate moment with a coherent representation of ‘my feelings’ about ‘that thing’*” (Craig, 2009b, p. 65).

**FIGURE 2:**
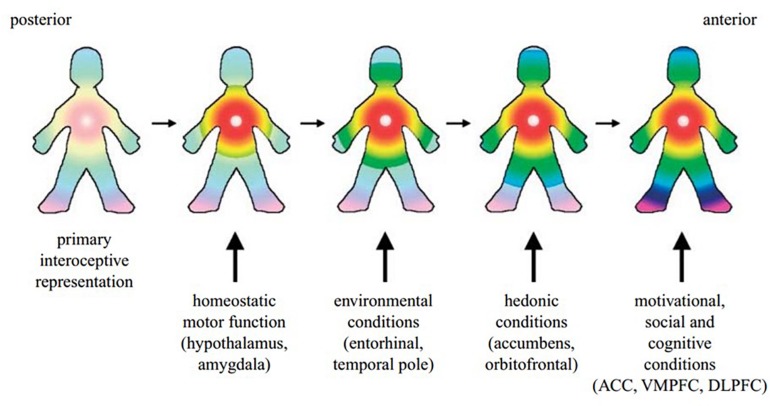
A cartoon illustrating how the hypothesized integration of salient activity progresses from the posterior insula (left) through the mid-insula to the anterior insula (right). The primary interoceptive representations of the distinct feelings from the body in the dorsal posterior insula provide a somatotopic foundation and a template for the construction of all feelings. It is anchored by the homeostatic effects of each feeling on cardiorespiratory function, as indicated by the focus of the colors in the chest. The salient homeostatic, environmental, hedonic, motivational, social and cognitive factors are progressively integrated by the indicated inputs. VMPFC, ventromedial prefrontal cortex; DLPFC, dorsolateral prefrontal cortex. Reprinted with permission from Craig (2009a), Philosophical Transactions of the Royal Society B: Biological Sciences, 364(1525), 1933–1942.

It has further been argued that homeostatic processes and interoceptive signals underpin the experience of self. The posterior to anterior re-representation of interoceptive sensation within the insula allows for the integration of the body’s homeostatic condition with exteroceptive sensory input, as well as information about the individual’s motivational and social context from other brain regions such as the ACC, hypothalamus, amygdala, ventral medial and dorsolateral prefrontal cortex and the ventral striatum (Craig, 2009a) (Figure 2). Such a perspective is supported by Critchley and Seth’s conclusion that the “*insular cortex supports a neural representation of changes in internal arousal states, and, within anterior insular cortex the re-representation of this information is proposed to underlie subjective emotional feelings and their abstraction to both the encoding of future risk and the experience of empathic feeling for others.*” (Critchley and Seth, 2012, p. 424).

Damasio (2010) similarly proposed that interoception plays a crucial role in generating a subjective sense of self. However, while Craig places the self firmly in the insula, Damasio cited a patient whose anterior insula was destroyed by a brain lesion but continued to exhibit all signs associated with feelings and awareness of self (Damasio et al., 2013). A possible reconciliation of these opposing views is that the neural substrate of feeling states is to be found first subcortically and then secondarily elaborated at a cortical level (e.g., in AIC and ACC) (Damasio et al., 2013; Solms, 2019).

Building on all this, an overarching model of how interoceptive processes produce subjective awareness has been presented by Craig. The foundation of this model is in the perception of neural interoceptive signals as sensations (Craig, 2010). These signals generate pain, temperature, itch, hunger, thirst, muscle burn or ache, joint ache, sensual touch, flush, visceral urgency, nausea, among other sensations (Craig, 2008). At any given moment, the pleasant or unpleasant quality of such interoceptive sensations imbues them with motivation for the individual to move toward or away from the source of the sensation, consciously or not, while causing reactive responses in the ANS (Craig, 2008, 2010). Craig defines this functional combination of interoceptive feelings and motivation, with autonomic sequelae, as “*homeostatic emotions*,” and likens them to Damasio’s “*background emotions*,” which Barrett calls “*core affect*” (Craig, 2008 citing Damasio, 1994; Russell and Barrett, 1999; Barrett et al., 2004).

Background emotions may be discerned through body posture, movement of the limbs, speed of motions, and animation of the face. One might use words such as “tense,” “edgy,” “discouraged” or “enthusiastic” as signifiers of such experience (Damasio, 1994). A similar approach is taken by Barrett, who contends that there is likely to be a core affective system which has the basic function of integrating sensation from the external world with interoceptive information. This integration generates “*a mental state that can be used to safely navigate the world, by predicting reward and threat, friend and foe*” (Barrett, 2011, p. 364).

Craig, Damasio and Barrett thus all propose that the underlying emotional experience within an individual is determined by the homeostatic management of their body’s physiology, influenced by the motivational state of the body with respect to these interoceptive sensations. The whole process is constantly engaged in reconciling the past with the present moment, on a physiological level within the individual’s body.

Interoception is, similarly, vitally involved in: self-awareness (Critchley et al., 2004; Craig, 2009b); feelings of conscious presence (Seth et al., 2011); the integration of cognition with emotion (Berntson et al., 2011; Gu et al., 2013b); and empathy (Singer et al., 2009; Gu et al., 2012, 2013a). Abnormalities in the perception of interoception have been linked to: anxiety (Paulus and Stein, 2010; Duquette, 2017); depression (Harshaw, 2015; Duquette, 2017); alexithymia (Herbert and Pollatos, 2012); eating disorders (Eshkevari et al., 2014); and depersonalization (Sedeno et al., 2014; Owens et al., 2015).

## Predictive Processing, Inference and Bayes’ Theorem

If we assume that the integration of interoceptive sensations with exteroception and top-down information underpins subjective experience, the all-important question for therapists is how does this process produce and maintain our patients’ views of themselves and the world? This question is best answered within predictive processing (Clark, 2016) where subjective experience is assumed to flow from top-down inferences that contextualize bottom up (bodily/interoceptive) sensations, while not assuming any sharp distinction between cognitive or non-cognitive processing (Seth and Friston, 2016).

As the brain does not have direct access to the world, it can only make sense of the individual’s internal and external environment (or world), by inferring the causes of sensation.^2^ The most interesting aspect of predictive processing theories is that they stress that our experience is largely dependent on what our brains “predict” or “expect” is happening, at any given moment, based (at least in part) on previous experience (Gu et al., 2013a; Seth, 2013; Barrett and Simmons, 2015; Seth and Friston, 2016; Ondobaka et al., 2017; Miller and Clark, 2018). Such expectations or beliefs are not necessarily conscious, nor available to awareness, for example, the “beliefs” we formed in infancy about comforting or fearful stimuli.

The predictive process that the brain uses to infer the causes of its sensory states can be described statistically by Bayes’ Theorem, which provides a principled way of describing how we test and update our hypotheses (also sometimes referred to as our implicit “priors,” “predictions,” “expectations” or “beliefs”) against the evidence supplied by our (interoceptive and exteroceptive) sensory organs, learning (and continually updating) the mostly likely cause for some particular incoming (set of) sensory information.^3^ As a “Bayesian observer,” the brain attempts to “know” about inner and outer experience by calling up a previously determined prediction (A) (also “prior”) that seems most likely to explain the current sensory evidence and then comparing that prediction with the actual incoming flow of interoceptive information (the observation X). If they don’t match, a “prediction error” occurs that may then be used to update the prior belief, creating a new (“posterior”) belief.^3^

This process of evaluating incoming sensation against prior belief is hypothesized to occur throughout the brain, in a hierarchical manner, i.e., through a step-wise process of activity, within neuroanatomic (hierarchical) levels of the brain. For ease of understanding, the terminology used: “lower” vs. “higher” in reference to this hierarchy, refers to areas that are more sensory bound as “lower,” while those that are more bound to prior beliefs or associative processes are labeled “higher.”

Predictive processing is well understood in vision (Rao and Ballard, 1999). For example, I am walking in the street and see a shape in my peripheral vision. The sensory input to my retina quickly matches a pattern that generates a hypothesis (belief) that it is a tiger. This is my “prior.” I look more carefully and see either that yes, indeed it is a tiger (hence no prediction error). Or perhaps I see that it is a large cat! This evidence (very different from my initial prediction) results in a large prediction error and a considerable revision of my original (mistaken) prior belief about a tiger – into the posterior belief that provides a much better, error minimizing, explanation for my sensations – namely, that what I am seeing is a cat.

## Free Energy and Entropy

As a prominent advocate and extender of predictive processing, Karl Friston’s innovative thinking culminates in the “free energy principle” (Friston et al., 2006; Friston, 2010, 2013) which asserts that in order to maintain homeostasis and survive all living organisms must avoid surprising states (i.e., free energy). Clearly, when applied to interoception, minimizing surprise is just another way of describing homeostasis (i.e., keeping interoceptive signals within a comfortable and familiar range). At a more general level, human beings don’t like surprise – or its mathematical average – namely, uncertainty (Edwards et al., 2012), which is equivalent to summed prediction error, i.e., the difference between what our brains predict and what our actual sensations are at any given point. Minimizing free energy is equivalent to resisting entropy (the tendency for a system to become disordered, dissipate decay and ultimately die). The beauty of the free energy principle is that it accounts mathematically for the inherent drives that biological organisms have toward allostasis and self-organization.

In effect, free energy can be thought of as the difference between the actual states of an organism and the states it “believes” (generally unconsciously) that it needs to be in for its adaptation, survival and reproductive success. When a prior belief doesn’t match the incoming sensory data, there is prediction error (i.e., uncertainty or free energy), which we can reduce by updating our beliefs (from a prior to an improved posterior belief) about the state of affairs in the world. In other words, updating our priors makes our predictions better explanations for sensation and thus minimizes prediction error. This process of updating priors is “perceptual inference.” Assuming that priors are generally encoded in higher levels of the brain’s hierarchies, this implies that prediction errors ascend the brain’s hierarchy to do the updating. However, all animals can instead act to change the world (and their own body) to make the sensations that they receive fit with their predictions. For this to happen, at the very bottom of the hierarchy (i.e., at the most sensory bound level) prediction errors descend to activate the effector organs to elicit motor reflexes. This is known as “active inference.” In our example of the supposed tiger in the street, the original belief that there is a tiger immediately invoked a higher level (learned) cognitive prior that tigers are not generally seen in suburbia. To resolve uncertainty about what is actually causing visual impressions, the brain predicts that it will foveate the supposed “Tiger.” These predictions about the (proprioceptive and exteroceptive) consequences of “looking over there” are then issued to the oculomotor system. In turn, the oculomotor system resolves proprioceptive prediction errors by using them to drive motor reflexes that point the eye to the predicted (i.e., intended) location. In essence, this is active inference, where prediction errors drive bodily changes to eliminate themselves and – in so doing – fulfill top-down predictions.

Predictions or beliefs can be straightforward, e.g., about how far our hand must move to reach a pen, or they may be highly abstract and refer to the social intentions of another human being. Some predictions will be innate and not subject to updating (such as homeostatic set points) but others are at least partly learned – many in infancy. Updateable priors include priors about policies/actions (e.g., priors about using a habit); priors about models of the world, and priors about beliefs within a generative model of the world. Importantly, some priors must govern the supposed reliability and salience of any given incoming sensation (these are priors about “precision” – discussed below).

If there is discordance between sensations and the expected cause of those sensations, surprise, uncertainty and free energy are ostensibly increased. Minimizing prediction error/free energy from moment to moment ensures that human organisms adapt and survive. In a perfect world, free energy minimization thus results in healthy, optimal functioning. However, the world isn’t perfect – it is predictably unpredictable.

Combining the biological imperative that humans are at equilibrium with their environment (internal and external) when free energy is minimized, together with the psychological assertion that human beings avoid pain and approach pleasure, Joffily and Coricelli elegantly link free energy, or uncertainty, to the valence of the emotional state. They suggest that if there is a rise in free energy, due to a mismatch between expectations and sensory input, there will be more inherent surprise or uncertainty, producing an emotion with negative valence. Examples are fear, disappointment and unhappiness. If there are prediction errors but the opposite is true and free energy is falling, then the valence of the resulting emotion is positive, such as for happiness or hope. Intriguingly, Joffily and Coricelli go further and suggest that fear can be distinguished from unhappiness because fear involves not only rising free energy (the organism is moving away from desired set points) but also includes the expectation that this rate of change will accelerate. Unhappiness, by contrast, involves only that free energy has risen but with no expectation that this change will get worse. For happiness the expectation is that the fall in free energy will accelerate, while for hope there is no such expectation (Joffily and Coricelli, 2013).

## Active Inference in the Interoceptive Domain

As outlined above, prediction errors may update perceptual priors (this is a case of changing the brain’s inner model to better fit the world). Alternatively, they may be resolved by descending to the brain stem and driving motor reflexes so that the animal acts on the world, which may serve to make the world a better fit for the prediction.

In the case of interoceptive prediction errors, descending prediction errors can enslave ANS reflexes (e.g., by raising heart rate in response to the perceived threat). Thus, in our tiger example, a high-level cognitive prior (learned or partly innate) is also immediately invoked that the viewer is in danger. This prior sets up prediction errors between the currently relaxed state of the body and the state it needs to be in to evade a predator. These descend to activate ANS reflexes and are eliminated by invoking high arousal. Prediction error is thus minimized by changing the body to better fit the world (Critchley and Seth, 2012; Gu et al., 2013a; Seth, 2013; Hyett et al., 2015; Seth and Friston, 2016; Critchley and Garfinkel, 2017). This process, known as “active inference,” is a special case of prediction error (or free energy) minimizing in the interoceptive domain; where action corresponds to autonomic regulation (Critchley and Seth, 2012; Seth, 2013; Pezzulo, 2014; Barrett and Simmons, 2015; Seth and Friston, 2016; Quadt et al., 2018). This circular signaling between body and brain, causing autonomic reactions, can serve as an important entry point into therapeutic change, as we discuss below.

The literature on reinforcement learning and the formation of “habits” is relevant here. It is proposed that reinforcement learning takes place in two ways. Model-based (goal-directed) earning is essentially Bayesian, whereby the learner has a model (e.g., a prior belief) that updates in the light of available evidence. In model-free (habitual) learning, on the other hand, “*through the process of sequence learning, action control becomes increasingly dependent on the history of previous actions and independent of environmental stimuli, to the point that, given some triggering event, the whole sequence of actions is expressed as an integrated unit*” or habit (Dezfouli and Balleine, 2012, p. 1038). Many empirical studies (in rodents and humans) show that, during reinforcement learning, behavior is initially goal-directed but then becomes habit-based. Importantly, it has been shown empirically that habits are insensitive to both changes in the context and changes in reinforcement (Dolan and Dayan, 2013). In other words, they persist inappropriately. In Friston’s words “*after the habit has been acquired, there is no opportunity for pragmatic policies. This means that although the behaviour is efficient in terms of reaction times, the habit has precluded exploitative behaviour*” (Friston et al., 2016, p. 874). In this way our patients’ emotional states can reinforce a style of interacting with the world (habits) on the basis of active inference processes gone awry and not in their best interest.

As a keen observer of the patient’s experience, the therapist is poised to support the patient in affecting change in the role that dysfunctional priors (all too often from model-free/habitual learning) play in their feeling experience, bodily reactivity, behavior and thinking processes. The therapist, alert to when the patient’s bodily reaction may be patterned along beliefs other than those realistically related to the present moment, can be the initial observer of these and invite the patient to actively attend to the sensory experiences of their body. Attention invokes the crucial role of “precision” in predictive processes.

## Interoceptive Inference and Subjective Experience – the Role of “Precision”

Within predictive processing theories it is well-understood that “prediction,” “prior” or “belief” do not denote a consciously held belief, but refer to activity occurring in the brain, which is assumed to encode probability distributions (i.e., subpersonal Bayesian beliefs). These distributions reflect the likelihood that a particular prior is a good explanation for the current sensory input that the brain is receiving (e.g., that “tiger” best explains the sensory pattern on my retina). There is uncertainty (variance), associated with any probability distribution. The inverse of this variance – known as “precision” – is the salience, confidence or reliability attached to a particular prior or prediction error (Friston, 2009). Within the brain, at any given moment, the attached precision (i.e., the relative reliability/salience) of the bottom-up sensation vs. the top-down prior belief is modulated by many factors (such as attention and motivation). The relative weight, i.e., the precision of the prior belief vs. the incoming sensory information is the determining factor in whether updating of a prior occurs. Prediction errors stemming from sensation that is precise (reliable) will update an imprecise prior. However, a highly precise prior (e.g., a historical prior or habit that has preverbal roots from infancy) may resist updating. For example, the prediction errors for danger invoked by the possibility of a tiger are highly precise and drive ANS as well as motor reflexes. Nevertheless, the prior for a tiger loose in a city is very imprecise (unlikely) and is easily updated to “cat.”

Attention is a key driver (or psychological homolog) of precision (e.g., Feldman and Friston, 2010; Edwards et al., 2012). Pezzulo (2014) uses an engaging story about a dark night, a creaking shutter and fears of an intruder (the imaginary bogeyman), to illustrate this. He notes that interoceptive sensory information is often afforded more attention than exteroception because it is commonly experienced as more certain by the individual, thus maintaining higher precision relative to other sources of sensation and consequently asserting a disproportionate effect in the inference process. Pezzulo describes how the resulting affective experience and the resulting physiological reactions might create a belief of immediate danger (from a bogeyman) in the middle of the night. Importantly for our purposes, he describes how shifts in precision due to unrecognized attentional imperatives can result in experiences and behavior that seem to reflect the reality of the moment, yet are actually the result of significant distortions (Pezzulo, 2014).

In our patients’ models of the world, precision dictates how strongly they hold to their priors, in spite of evidence to the contrary. Patients suffer where they rely on highly over-learned and thus very precise priors which do not reflect the truth but are difficult to update, as they have gained relative strength with repetition over time and have become habits that are insensitive to changes in context or reinforcement. For example, for an anxious person at times of fearful distress instigated by perceived threat, the default prior “danger” will be afforded higher precision. As a result, prediction errors signaling that they are actually safe don’t update this prior, as the individual fails to attend to, and thus increase the precision of, disconfirming evidence in the here and now. Crucially, these prediction errors can be resolved instead by changing the body so that it fits the habitual prior (e.g., by raising heart rate to fit the prior for threat). In other words, interoceptive prediction errors instigate the very autonomic reactions that drive the ANS into inappropriate arousal (freeze, flight, fight) in order to confirm the habitual prior. This high bodily arousal will be experienced as fear, as the emotional state updates to fit the incoming information of arousal. From the perspective of the patient’s emotions, this illustrates the classic James-Lange contention that we feel fear when we receive peripheral information from the body.

What is striking here, for psychotherapeutic treatment, is that the *expected* interoceptive arousal state that corresponds to fear – such as muscle tension, heart rate increases, or hormonal response – is actually being *produced* within the body in response to the initial highly precise prior of perceived threat, although such a threat does not actually exist at that moment. Such conceptualizations explain the observations at the beginning in this paper that a patient will be certain that she is scared only because her heart is beating faster. This contention can now be seen as circular causality between the brain and the body, provoked by active inference. Interoceptive prediction errors have descended to activate ANS reflexes that affect the body by raising heart rate. Sensation that has been created by ANS activity then returns up the hierarchy to the brain and verifies fear as the person’s “*value-based choice about the internal state of [their] body*” (Gu and FitzGerald, 2014, p. 269). In other words, the high precision of the habitual/default prior for danger unfortunately specifies that fear is indeed a predicted and familiar state for the individual to be in, which itself reinforces the precision of the prior rather than there being any attention to disconfirming evidence. Sadly, the brain returns the body to a state of fearful arousal, simply because this is the expected state, ostensibly with free energy lower overall as there is less uncertainty (Friston, 2010; Clark, 2016; Ondobaka et al., 2017). Such moments – when precise prior expectation cause ANS reaction and thus bring about the perceived confirmation of the original expectation – are important points of therapeutic access, which we will address in detail below.

Some priors are obviously more resistant to updating from sensory evidence than others. Yon and colleagues point out that if the brain were actually an “*ideal scientist*,” as predictive processing explanations imply, then a hypothesis about the causes of sensation would simply be compared to the evidence and the brain would update this hypothesis accordingly. But in real life priors are often resistant to disconfirmation, either as a result of evolutionary pressures (as in the case of homeostatic set points) or through developmental processes, creating a situation where the brain acts more like a “*stubborn scientist*” (Yon et al., 2019, citing Bruineberg et al., 2018). This characterization highlights how important it is to investigate and address possible sources of resistant prior beliefs or habits. All psychological treatments have some process orientation that supports the instigation and testing of hypotheses by their patients.

For the purposes of this paper, we focus on infancy as a vital time for the inferential processing of experience into beliefs about self and others, habits, as well as the linking of emotion and interoceptive processes and experience, within the caretaking relationship. The psychological literature centers on the importance of attachment in how an individual will react to strong emotions (e.g., Stern, 1985; Bowlby, 1988; Siegel, 1999). For example, the phrase “*implicit relational knowing*,” used by Lyons-Ruth et al. (1998), Morgan et al. (1998), and Stern et al. (1998), places the development of procedural knowledge concerning both interactive processes and affective experience within the relational interactions between caretaker and infant. Such caregiver-child interactions are especially important with respect to periods of strong arousal, which is considered to be the burgeoning experiential element of early emotions (Schore, 2003).

It has been proposed that physical contact with caretakers in infancy acts as an early homeostatic regulator, supporting the development of the immature nervous system (Fotopoulou and Tsakiris, 2017). Furthermore, it is argued that through the quality of caregivers’ understanding of our body’s needs, coincident with our nascent inferential processing of interactions with them and with our inner and outer environments, our brains develop early (unconscious) “prior beliefs” about the causes of our sensory states. A child’s brain, for example, forms beliefs about what situations are comforting or fearful through caregivers’ effective (or ineffective) provision of necessary resources. Such preverbal experiences (with subcortical representations that may be more salient – i.e., have higher precision – than any later cortical elaboration) strongly influence our physiological regulatory processes and become imbued with motivational significance, thus forming the basis of our subjective emotional experience and our interactions in the world. Activities within an attachment relationship that settle the infant’s nervous system occur following successful interactions between the infant and the primary caretaker. In this case, interoceptive signals facilitating homeostatic balance within the emerging predictive system of the infant then support a sense of physiologic stability and overall well-being (Fotopoulou and Tsakiris, 2017).

While addressing the importance of physical contact and interpersonal interactions with caregivers on the development of the self, Fotopoulou and Tsakiris (2017) link such bodily based interactions with the development of early “*mentalization of interoception*.” Importantly they note that it is the direct proximity, style of contact and care of the infant’s homeostatic needs which are important progenitors of experience. As such interactions become more complex, the growing child can “*build increasingly more sophisticated models of their own interoceptive states, as well as strategies for minimizing free energy in the interoceptive system*” (Fotopoulou and Tsakiris, 2017, p. 17).

Commenting on the originally subcortical nature of emotion (which is subsequently elaborated in the cortex), Solms (2019, p. 13) makes the important point that “*subcortical memory traces cannot be retrieved in the form of images for the simple reason that they do not consist in cortical mappings of the sensory-motor surface organs*.” This has the crucial implication that feelings states (emotions) that have been acquired as habit sequences in infancy may lack cortical expression. It is our contention that these may, therefore, only be accessible through the bodily (interoceptive) representations that accompany them. Daw has suggested that “*hierarchical reinforcement learning decompose a multistep decision problem into a nested set of choices at different levels of temporal abstraction*.” He proposes that lower-level choices are essentially “*model-free: stereotyped behavioral sequences, like a tennis serve or a dance move*” (Daw, 2015, p. 13750). If this is so, then our patients’ precise priors (that appear unavailable to updating) may be characterized as low-level habits or routines to which the patient has little conscious access, other than that they create interoceptive (ANS) sensations. It is therefore by attending to these interoceptive sensations, in the safety of the therapeutic relationship, and by challenging their relevance to the here and now, that we can increase the precision of disconfirming evidence and hopefully move the patient from a model-free reaction to one that is model-based, in which current evidence can be evaluated and can update the model.

## Implications for Psychotherapy

As discussed above, it is generally accepted that emotion is always accompanied by interoceptive sensations within the body that signal what is motivationally salient to the individual. These sensations can become conscious “feelings,” which we define here as emotions that are known and/or verbalized. While not all emotion reaches awareness in the form of feeling, each psychological school of thought has cogent theoretical reasons for why it is therapeutic for the patient to bring emotions into subjective awareness. Our focus here is to propose that attending to interoception is crucial in this context.

Emotion, defined as sensation with coincident motivation and resulting autonomic sequelae (Strigo and Craig, 2016, citing Rolls, 1999) can be described through the processing of incoming interoceptive sensation that is integrated with concurrent exteroceptive sensation (Seth and Friston, 2016). The process by which this occurs, i.e., the “generative model” that is used in the current moment (with its priors and precisions at every level of the hierarchy), will tend to follow a pattern (i.e., a habit), that often represents a view of the world and the self that was experienced within the early caretaking relationship. Such reactions are not consciously remembered *per se*, but exist as a set of bodily felt sensations that persist due to the salience (the precision) that was afforded to the various sensations in these early relationships. For example, if the infant was not responded to with consistent signaling of safety/certainty by caregivers – communicated at the bodily level – the prior for threat will be highly precise. That adult individual will tend to stay in a state of increasing uncertainty about how others will respond and may be unable to take account of disconfirming evidence. There follows the crucial insight that this uncertainty/anxiety may actually be a highly familiar state to the individual. The pernicious potential effect is that the brain will tend to accept this state as that to which it should seek to return the body, in order to minimize free energy, despite the fact that this state is represented by the patient as distressing. For psychotherapeutic treatment, the implications of interoceptive inference are thus profound. Our task is to revise such familiar, but dysfunctional, priors (habits) to which the patient tends to return. However, the power (i.e., precision) of such priors highlights the early experiences of the body, which may not have an explicit conceptual component although they have bodily/emotional precisions that are very important for determining the individual’s current state. Our proposal is that acknowledging the influence of bodily sensation on the processes of mind, and creating a window into the influence of the body and sensory experience on emotional states and cognitions, allows therapists a much wider range of interventions based on the body than when engaging relationally with our patients in “just” talk therapy.

To illustrate this, it is important to remember that within a predictive processing view emotions and feelings are always hypotheses that provide the best explanation for the many interoceptive and exteroceptive cues which have to be explained. For example, the (high-level, conscious) hypothesis “I am anxious” may be the best available explanation for a breadth of visual, auditory, somatosensory and (crucially) autonomic sensations that are currently in play. Therapists can recognize (rather readily) that there may be several other possible explanations, yet patients are likely to reference a habitual explanation for the stimuli that are commonly salient to them, often creating a “story” regarding their meaning, even if such responses are sub-optimal or create dysfunction. In determining the point at which an intervention can be implemented therapeutically, we make an important distinction between: (i) “active interoceptive inference” in terms of a pre-reflective, subpersonal assimilation of interoceptive cues that contextualizes our autonomic reflexes and reactions to emotionally salient cues; and (ii) the personal, reflective or propositional inference involved in “mentalizing interoception,” that raises emotions into subjective awareness (as feelings) and thus involves explicating the content of active (interoceptive) inference that is available to conscious awareness (Fotopoulou and Tsakiris, 2017; Ondobaka et al., 2017).

To reiterate, bodily-based experiences will often over-determine current perceptual content (i.e., emotional priors). There may be a habitual and sub-optimal prior (generally unconscious) that connects some harmless stimulus to a sub-personal interoceptive inference, such that the body responds as if there is a threat, with ANS sequelae. At this juncture, however, if the patient is to engage in the selection of the emotional state that is a better explanation for the sensory evidence at hand, and reality in the present moment, s/he has to have more than one hypothesis (prior) available. In other words, s/he has to be able to select one emotional hypothesis over another, particularly in times of emotional distress, in order, for example, to distinguish “I am just excited” from “I am anxious.” The therapist cannot draw attention to the low-level prior that set the bodily/emotional reaction into play (because this is subpersonal, or unconscious). However, by drawing attention to the patient’s physical reaction (the ANS sequelae) – which is available to awareness – they can heighten the precision of the interoceptive evidence, thereby potentially allowing the inferential process to affect the habitual prior. This creates an opportunity to explore new priors, i.e., alternative explanations for these (now attended) bodily sensations, thus enabling interoception to be consciously mentalized. Achieving this desirable “mentalizing of interoception” necessarily requires elevating (low-level) subpersonal emotional experience to a higher, reflective level that, in turn, requires attending to pre-reflective interoceptive inference i.e., attending to current interoceptive sensations (Barrett et al., 2016; Hoemann et al., 2017). This mentalizing ability can, however, only emerge if we first attend to (and thus increase the precision of) interoceptive sensations.

Given that predictive processing theories indicate that to alter habitual ANS reflexes requires a change in the precision of the sensory evidence, to achieve this within the therapeutic interaction, requires the patient’s active attention to current bodily sensations, for example, the sensations associated with their body in the chair and the feeling of the ground beneath their feet. The mentalizing of interoception ostensibly starts as an “imaginative” process of exploring bodily sensation. While, strictly speaking, this also involves somatosensation, if the patient attends and tries to receive, assess, and appraise the embodied nature of all the sensation perceived, the result will necessarily involve interoceptive appraisal (Farb et al., 2015).

Focused attention increases the precision of current bodily sensations (thus generating ascending interoceptive prediction errors). There is now increasing uncertainty. This at first leads to an increase in the intensity of the experienced feeling, which the patient will find unpleasant and to which the therapist must consequently respond. The therapists’ persistent observation and enquiry into the patient’s experienced state of their body is vital at this juncture. Observing physical expressions such as eye contact, facial and body movements, prosody of speech, muscle tension, will facilitate a qualitative assessment of changes in the state of the ANS (Schore, 2012).

Furthermore, the therapist must remain alert to many other variables in the therapeutic process which affect emotional valence and which can cause change from positive to negative valence (representing dynamic changes in free energy), without the patient necessarily being aware. For example, sudden changes in the manner in which patients express feelings verbally (less spontaneous verbalizations, word choice, prosody), or alterations in the patient’s physical response (more body tension, sitting forward/back, folding up their legs and arms) could indicate changing emotional valence within the patient and rising or falling free energy. Pointing out bodily changes to the patient, and encouraging them to reflect on what the therapist observes, can bring to the fore contextual aspects of the present moment that are stimulating increasing uncertainty in the patient but were unrecognized by her. We propose that these physical expressions are linked, ultimately, to early beliefs and relational expectations, which are not recognized cognitively but are being expressed bodily. It is our contention that when these come to awareness in the patient, this provides an important element that supports change, in any therapeutic process.

Uncertainty is constantly being evaluated across multiple hierarchical levels in the brain at any moment, influencing experience and behavior. As the therapist models recognition of bodily changes to the patient and enquires about emotional valence, and the change of valence in either direction, the patient can better take a reflective position regarding their bodily-based responses to their internal and external environment and the attached uncertainty. This is a functional benefit of mentalizing interoception. A unique blend of possible insight is available to the patient at such times – into affective experience, cognitions and current moment perspective – which also invite a process of change which the patient can implement when they are no longer in therapeutic session.

As the patient tries to discern the inherent faults of their models of the world, the therapeutic relationship should offer a “safe haven,” otherwise, a sense of threat may irreparably impact the freedom the patient requires to explore. The safety of the therapeutic relationship is paramount and must be attended to, explicitly and implicitly, between therapist and patient. The therapeutic attachment relationship in widely thought to be important in supporting not only the perception of safety but also to facilitate change (e.g., Cozolino, 2002; Wallin, 2007; Siegel, 2010). While a detailed discussion is beyond the scope of this paper, we will focus on an element of the therapeutic relationship, “the real relationship,” which has specific aspects that can be operationalized regarding the therapeutic relationship, the treatment environment, and the predictive process aspects of the mentalizing of interoception. The real relationship, is defined as the non-transference part of the relationship (Gelso, 2009), concerning the authentic, genuine and realistic aspects of the relationship between therapist and patient (Duquette, 2010). We briefly outline below what the real relationship brings to the process.

The consistent presence of the therapist, experienced repeatedly in times of uncertainty creates a history of moving together within the therapist/patient dyad. And hopefully, such times resolve for the patient with an increasing sense of verifiable safety amidst open vulnerability. Such history “*encourages the patient to move ‘deeper’ into the chaos experienced at any given moment*” (Duquette, 2010, p. 141). The experienced authentic, reality based, qualities of the relationship also presents a space that is distinctly different, and not laden with the expectancies that occur with people the patient knows (Morgan et al., 1998). Ultimately, as such elements of the relationship lessen uncertainty in the therapeutic process, thus decreasing prediction error, persistently supporting a gradient of decreasing free energy.

All schools of psychotherapeutic treatment must address their patients’ persistent prior beliefs, that are manifested in the form of cognitions or behaviors that stem from inaccurate priors that persist in the face of a different current reality. Therapists use every available resource to encourage the patient to become a flexible scientist and test their strongly held hypotheses against a different current reality. How the therapist will choose to support the shifting of priors, using the direction of attention, will depend upon the form of relational interaction delineated by their particular protocol. For example, in Acceptance and Commitment Therapy (ACT) the identification of old beliefs about the self will be paramount, together with means to direct attention away from these toward disconfirming evidence. In cognitive behavioral therapy (CBT) the identification of cognitions is key, with attention being directed to unlinking the associated reactive behaviors. The common experience of transference can also be interpreted within a predictive framework – as a process that molds the patient’s past priors onto the present relationship – while counter-transference is the functional equivalent for the therapist. The various therapeutic schools will view the outcome of predictive processes (including transference reactions) through the lens of their own philosophy but all therapists will be better equipped if they recognize the inherent difficulty that the patient has in trying to be an impartial Bayesian scientist with respect to the sensory data of their inner and outer world.

## Discussion of Clinical Vignette

The material and concepts outlined above inform the case study contained in Box 3. [Written informed consent was obtained from all mentioned individuals for the publication of this case report/case description, no identifiable information is included and pseudonyms were used]. Elements of Molly’s clinical interactions with the therapist illustrate how the neuroscientific ideas described above are useful in psychotherapeutic treatment.

**BOX 3 Clinical case study.**

[Written informed consent for the publication of this case study was obtained from all the individuals mentioned. No identifiable information is included. Pseudonyms are used throughout.]

Molly was 20 years old when she entered long term individual and group therapy. She made an appointment because her sibling who was in treatment suggested she do so, “If you need someone to talk to.” Molly could only express a sense that somehow “things just had to be different, life shouldn’t be this hard.” When Molly was a year old, her mother was psychiatrically hospitalized for major depression for several weeks and was thereafter often debilitated. Molly was considered bright and independent by her family, which was important to her sense of herself, an example she remembered was that by the age of seven she would travel miles away from their suburban home on her bicycle, only returning in the evening. At presentation she could describe that she was readily upset by small problems, had difficulty in relationships as she now saw herself as “inadequate and needy,” feeling constant self-criticism about her interactions with others. She complained of often not falling asleep due to ruminative thinking and having the sense “like something bad was going to happen” as she awoke each morning.

She rarely sat still, a leg jiggling, shifting in her seat, moving her hands. If asked what she felt in her body, she looked perplexed and said, “Nothing.” Although she expressed little insight into her emotional or physical state, she was quick to tears when touched by strong feelings, and would tense her facial muscles and throat to limit their expression. Her answers were often content-based and lacked contextual depth. However, with a clear sense of empathy for other’s experiences, Molly rarely was critical of others, but of herself in most instances.

In an individual session early in her therapy, Molly’s constant motion was addressed by her therapist. “You seem to be constantly on the move, any feelings that you are aware of?” She recognized she did “move a bit but if I don’t I will be bored.” When asked what “bored” meant, she could only say it was like, “an empty place, that just isn’t good to be in.” Her therapist then asked Molly if she could sit entirely still and Molly immediately flatly insisted, “No that would just be too uncomfortable.” Quizzically the therapist asked, “Uncomfortable? Sitting still is more uncomfortable than moving your legs, hands, and shifting in your chair so often?” “Sitting still is just uncomfortable, like painful uncomfortable for me, maybe not you, but for me it is.” Does it feel as if the painful sense is in your body somewhere, or does it feel as if something painful will come from outside your body if you sit still?” Molly paused for a minute, “Hmmm, well now that you put it that way…I know I do feel uncomfortable in my body, I don’t know but maybe it’s that thing you call feelings? I just never sit still with people and it is just safer all around.”

Her therapist offered, “How about you give it a try here, with me. How about you put your feet on the floor and your hands in your lap, just for 30 seconds now?” Molly resisted through a few rounds of interaction, then skeptically placed her feet on the ground, and put her hands in her lap. She didn’t make eye contact and she held her body rigidly in place, but her breathing slowed gradually as her eyes softened and began to well with tears. “Any idea of what your tears are about?” her therapist asked. After a pause, Molly said, “It just feels like in the world there is too much that will hurt me, and I have to keep away and keep moving to be safe.” Her therapist invited her to make eye contact, take a deeper breath, while also talking about the obvious elements of safety of the moment and how she, the therapist, was alert to any possible dangers for Molly. Slowly Molly raised her eyes, took a slightly deeper breath as tears fell more readily from her eyes. She said slowly, “It just feels like I have this pressure in my head, like at my forehead, like all my feelings are bound up there.” Her body had become less tense, her voice softening. Her activity level didn’t increase again throughout the remainder of the session, exhibiting a deepening emotional involvement with her therapist.

Molly drove an old car and she could make any necessary repairs, which were frequent. One day she repaired her car alone on a city street in a dangerous area, rather than asking anyone to help her fix it or get it towed to a safer place. She came to group directly after that repair, with dark grease on her hands and her clothes disheveled. A group member, visibly upset, said “How could you just start working on your car in such a dangerous part of the city, and alone, too!” Molly answered caustically, “And what was I supposed to do? I had no choice, I had to get here, I don’t need to ask anyone for help, that’s how I could work on it on the street. I don’t care where the street is!” The other patient asked, “Did you even think about asking anyone for help when your car broke down?” “No, I’ve never asked anyone for help when I can take care of the problem.” With a softer voice, the other patient said, “But you were on a street in the city alone, you could have been in a lot of danger, just having someone nearby would have been a help, wouldn’t it?” Molly became more physically agitated at that interchange. “I don’t care that I was alone, I can take care of myself just fine, that’s just the way you do things, no one is there to help with problems, they just add to them, you just don’t understand.” With that Molly sat back in her chair, arms and legs crossed with a deep scowl on her face and looked at the carpet.

Her therapist spoke to her next, saying softly “Any idea of what you feel?”

“They’re an idiot, that’s what I feel!”

“Well, Molly, “They’re an idiot” is not a feeling—any emotional feeling, hurt, scared, angry?”

“No, well, maybe pissed!”

“Hmmm, your voice has tears in it, does that fit with pissed?”

“I don’t know, they can be as critical as they want, I take care of what I have to. However, I have to take care of it, and I don’t need anyone’s help.”

“Right now you appear to need some kind of help, you have tears in your eyes and your voice, and a frown on your face, your body is folded up tight.”

“I’m just pissed.” Molly said this with tears welling in her eyes, her eyes focused on the rug in the room, and her body tensing more.

Molly continued to frown, while tears spilling down her face, which she wiped away with force. She was invited to unfold her arms, and to set her feet on the floor. She did this, reluctantly, her eyes remaining focused on the rug in the center of the room. The other patients looked concerned and at a loss as to how to approach Molly as she interpreted their compassion as judgment regarding her choices.

The therapist believed that Molly felt not only threatened by the other’s opinion but also by the compassion inherently expressed in the group’s stated concerns, which was disorienting as it was unfamiliar to her. To manage this feeling of threat, Molly had withdrawn from emotional contact with others and her body was shoring up her defensive position, both against interaction with others and self-experience. She believed the other misunderstood her independence, essentially belittling it when Molly wore it somewhat as a badge of honor. She could not access any sensation in her body, and was unable to think through any other possible explanations regarding her experience or actions and others’ intentions. Her therapist recognized that Molly had implemented the only action plan she had known throughout her life, while repairing her car, and was continuing to do so in the therapy session, addressing only the content of the issue in front of her. As her therapist recognized Molly’s body was highly reactive with fear, she understood that questioning Molly directly regarding her emotional state would only draw Molly’s bright brain into the answer, forcing Molly to create a “story” to explain her beliefs about her choice to repair the car, other’s intentions, and insist that she needed no further help from any others.

Her therapist asked, “What do you feel in your body right now?”

“Nothing…maybe some tightness”

“Can you say where the tightness is most?”

“All I know is I can feel that ball of tightness up in my head, here, near the front of my forehead.” Molly ducked her head further into her chest, eyes downcast.

“So, there is tightness. Does it feel like this tightness is trying to keep something inside, or keep it outside?”

Molly considered the question for a while and then answered hesitantly, “Inside?”

“Why would you need to keep something inside?”

“I often have this feeling in my head, like there is something physically bound up in there. Maybe it is feelings but it sure seems physical to me. I’ve always felt like this if there was upset anywhere, it just takes me into myself, where I know I’m safe.” Molly’s voice softened a little.

(“You say it may be feelings bound up in there. So, if those feelings moved, or were felt, it wouldn’t be safe for you? What could be the danger?”

“If the feelings moved others would be able to see them. And if they see them, they would react in a way that wouldn’t be good. It never has been good.” Molly’s voice sounded less tense and reactive with this answer.

“You would be vulnerable if they saw your feelings, eh? Your voice is changing now, can you feel any other sensation now?”

“I can feel my shoulders are tight and my legs want to run.”

As the therapist was interacting with Molly, she noted that not only had her voice changed but the pressure in her speech was less. She would glance at the therapist, and she appeared almost curious about the questions. At other points in Molly’s therapy, in the group, she had used an intervention which she considered at this juncture, always with Molly’s explicit agreement. The therapist would offer some words that Molly might say about her experience, and some simple physical actions to make with the words that amplified their meaning. And with the other patients’ agreement, Molly would express herself in the other patients’ direction, to facilitate a sense of relational interaction, and evidence the other holds no malice. Such interventions had helped Molly decrease her habitual response, by increasing her felt sense of her body, supporting her increased awareness of what emotion she was feeling in the moment.

The therapist asked Molly if she was willing to “try something to help her continue moving out of that bound up place,” to which Molly said “yes.” She invited Molly to sit out further in her chair and look at the patient who had addressed her earlier in the session.

“How about you say to her: “You don’t know what it is to not have help!” As loudly as you can.”

Molly began hesitantly, barely able to keep eye contact, but gamely trying to do so. With encouragement Molly’s voice became louder with each attempt. Soon she was saying the phrase very loudly, assertively and with direct eye contact. She then became quiet and tears began to flow readily down her face, the frown gone and her eyes much more expressive. All the members of her group were looking at her with encouragement, even the person at whom she was yelling. The tension in Molly’s body lessened visibly.

“Can you feel any more sensations now?” Molly replied with a wry chuckle as she motioned across the room, “My eyes feel less tight, and she doesn’t look like she did a while ago. I don’t know why but my head doesn’t have that bound up feeling, my legs feel really tingly, and my chest – it feels like there is something moving in it. And I feel a deep pain there.” She sat back with her eyes softening more, appearing to consider something carefully inside herself. “Well, the feeling that there isn’t any help has lessened, and I don’t feel so alone, but that tightness in my forehead is a lot less and some sort of feeling in my chest is really strong now. I think it feels like my heart has a place in my chest now? Which is good I know, but wow, does it hurt, too.”)

Within Molly’s history there are important clues from her early life that foreshadow her difficulties with physiologic and emotional regulation. Her mother is a highly inconsistent presence during her first year and likely longer, as Molly describes that her family viewed her as independent from an early age. There is evidence of homeostatic disruption: her sense that “something bad was going to happen” as she awoke; her body either over-reactive (moving all the time) or under-reactive (sudden bursts of tears); and her strong sense of discomfort in her body; and that her needs will be seen as an imposition on others. Such symptoms echo the premise that homeostatic processes are “*dependent on embodied interactions with other bodies*” (Fotopoulou and Tsakiris, 2017, p. 13).

The initial statement made by Molly, “*Things just had to be different, life shouldn’t be this hard*,” is pertinent. Habitual behaviors are leading to poor outcomes as she enters adulthood, and she can’t generate alternative hypotheses. She recognizes that she can’t successfully problem-solve even the minor issues in her life and reports her view of herself as “*inadequate and needy*,” which causes problems in interactions with others. Such persistent negative expectations create a snowball effect within Bayesian processing for patients with depressive disorders (Barrett et al., 2016). Such patients are not able to appraise disconfirming evidence adequately, either by discounting its credibility, or by seeing it as the exception rather than the rule, Molly’s ruminations presenting evidence of only her original hypotheses and beliefs (Rief et al., 2015; Kube et al., 2017, 2019).

Considering mentalization from the perspective of intentional mental states (Bateman and Fonagy, 2012), Molly’s initial inability to use language to express her emotions – only being able to know that life “*should*” be different, and her fear or despair is “*something bad*” that might happen – is indicative of mentalization in that form. The possible importance of her mother’s absence due to hospitalization and depressive symptoms, is indicated by Fotopoulou and Tsakiris (2017, p. 17) claim that “*the origins of all mentalization processes are not only embodied but also by necessity involve other people’s bodies, their physical presence, proximity, contact and most importantly, their homeostatically relevant actions*.” It appears that Molly’s temperament, physicality and the actions of other caretakers may have helped her push through such early deprivation. However, ultimately the effect of the impaired regulatory processes that she would have experienced with her mother’s long absences in her infancy are evidenced in her symptoms in each domain.

Early in the session, as the therapist enquires about Molly’s constant physical movement. She is initially unable to be still or to respond in a reflective manner and she expresses a strong sense of threat. Stillness would only leave her in “*an empty place*,” “*painfully uncomfortable for me*.” The interceptive sensation from her body simply reinforces Molly’s prior beliefs and fearful state. She cannot intentionally pursue any deliberation. At this juncture the therapist offers an alternative view. She encourages Molly to imagine whether she feels the pain inside or outside her body. When Molly begins to take part in this cooperative narrative about her discomfort, the therapist puts herself forward as a safe space in which to try out something new by inviting Molly to test her negative expectation “*here with me*.” The therapist encourages her to look for new evidence by making eye contact with her, while stressing “*the obvious elements of safety of the moment*.” In several ways the therapist thus scaffolds Molly’s efforts to mentalize her interoceptive experience, ultimately resulting in a noticeable difference in the activation of Molly’s body, with deeper breathing and tears.

Molly’s reactions to the therapist’s interventions relate directly to how the body can be supported within relational interactions, allocating resources more effectively allostatically, thus effectively altering the ANS reactivity that stems from suboptimal habitual priors. The physical changes observed – the slowing of her breathing, increased eye contact and lessened bodily tension, evidence a shift in Molly’s autonomic state from a high sympathetically driven state toward a state with more parasympathetic control (Zautra et al., 2010: Vlemincx et al., 2015; Strigo and Craig, 2016) and indicate an updating of priors.

Attending to sensory experience from a position of observation, without judgment, allows the individual’s higher-order cognitive processes to shift into state of more open consideration and observation (Farb et al., 2015). This permits a new flexibility, which can facilitate awareness of interoceptive sensations, to which the patient may not habitually attend, and which may promote positive experiences and lessen the individual’s automatic return to cognitive elaboration or “stories” (Fogel, 2009; Farb et al., 2015). Ultimately, there will be a decrease in the general energy output (i.e., free energy) that a person may typically spend on self-regulation, especially if they employ active inferential processes (i.e., firing up ANS reactivity), in which they are effectively trying to change their own bodies to fit the dysfunctional habitual prior to which the brain continually seeks to return them. This can be seen in Molly’s original assertion that “*I have to keep moving to be safe*” which finally shifts to less tension. Deliberate intention is required to move into such a more observational mode with respect to experience. Initially such a relaxation of energy may feel difficult for the patient, due to the automatic nature of the habitual prior. However, with practice, the sense of relief that can follow a change from lower to higher free energy (Joffily and Coricelli, 2013) can positively encourage the patient. Notably, Molly’s activity level did not increase again throughout the remained of her first session.

During the subsequent interaction with another group member, Molly’s habitual prior is voiced that others cannot be counted on, and must be critical of her. She became very agitated and after dismissing the other as “*an idiot*,” she said she was “*pissed*.” However, her tears and physical agitation were physical signals that she was not likely to be referencing the current interoceptive processes that were instigating feelings. When asked, why she was feeling what she claimed she felt, Molly further elaborated on the ruminative material (“*pissed*”), as the precision afforded to such beliefs severely limited any possible awareness of other options. As the therapist inquired into her bodily state, she helped Molly place her attention on her reported “*tightness*” and decrease her habitual ruminations. She understood that Molly couldn’t, at that moment, question the content of what the other (compassionate) patient had said to her. Instead, the therapist began drawing attention to the sequelae of her (overdetermined) sympathetic nervous system reaction of the moment, noted as tension by Molly, asking “*What do you feel in your body right now?*”

The therapist began with Molly’s word choice (“*tightness*” and “*bound up*”) but increasingly expanded the field of options (“*vulnerable*” and “*danger*|”). As she again helped Molly to direct her attention to different aspects of the experience, this supported Molly in gaining control of her attentional resources. This is crucial in making her interoceptive sensations more precise (e.g., “*Can you say where the tightness is most*?”). While this initially increased the strength of the affective feeling (as evidenced by Molly ducking her head and insisting that she has to withdraw into herself, “*where I know I’m safe*”), disconfirming evidence can gain precision by this path and new alternative priors can be considered. At this point the real relationship between the therapist and Molly is an anchoring element. The manner in which the therapist readily accepts Molly’s description at first but continues to question, as well as the inflection of her voice (“*You would be vulnerable if they saw your feelings, eh*?”), reflect that she and Molly have been through such moments before. Molly’s decreasing tension implies a change in the experienced emotional valence to a more positive level (even though she is uncertain of the outcome), implying increasing confidence that they have made it through together before to where the therapist is leading, without encountering the dangers she is habitually expecting at that moment.

As Molly continued to engage, her voice became less reactive, and she even expressed curiosity. As the therapist observed Molly’s increasing self-reflective stance, she decided it would be clinically helpful to invite Molly to express her experience to others in ways that lessened her sense of aloneness, speaking directly to the other patients, who verbalized their willingness.

The intervention with the other patient in the group encouraged Molly to address her prior beliefs that she was unsafe in interaction with others. She was helped to shift from the position of a “stubborn scientist” to that of a Bayesian observer. The physical changes in her body, as well as the increase in her verbal deliberations about the possible implications of such experience in the moment, is evidence of changes in the precisions of sensations throughout the hierarchy which activated her body and had previously prevented the evaluation of new evidence. Ultimately, at that point she could recognize others’ presence with her and allow such presence to be helpful. While there would have to be many more episodes of such experimental trials, as precisions develop a life of their own, Molly was able to acknowledge aloud a sense of vulnerability and “*deep pain*.” Her feelings could be witnessed by others, which she recognized as safe and necessary, indicating a more emotionally open and deliberative stance. Molly was then able to make a profound statement about the embodied experience of her own emotion – as if her heart had gained space in her chest, which was experienced as both somewhat painful yet also soothing.

Molly’s response to the therapist’s recommendation to engage with the other patients also highlights various findings in neuroscience research. Molly’s voice became more assertive with each attempt and she was able to direct her eye contact purposefully. Eye contact has been found to support attention to subjective experience and increase the accuracy of emotional report about interoceptive experience (Baltazar et al., 2014). Molly used more expressive language (“*I don’t feel so alone*”) to describe her feeling state (i.e., increasing the precision of interoceptive sensation and prediction errors, to counter over-precise priors). Allowing others to witness her tears is indicative of lessening fear in Molly, reflecting a changing emotional valence for her, and likely falling free energy. Making eye contact, breathing deeper and crying openly is proof that she was experiencing a change in emotional valence, suggestive of an increasing positive valence, as she became more hopeful. Such behavior and the resulting interactions between Molly and her therapist became possible, as free energy decreased, lessening the need for the highly defensive behavior exhibited before. Changes in her affective experience as Molly sat up straighter in her seat reflect Ceunen et al. (2014) finding that an upright posture is associated with more positive affect than slouching, possibly because an upright stance expresses pride or power. During the intervention in the group, there was an obvious shift in Molly’s ANS reactivity, as evidenced by her chuckle and less bodily tension (Ceunen et al., 2014). Payne et al. (2015) assert that diverting the patient’s attention temporarily to a physical experience that gives them a sense of safety can mitigate extremes in autonomic reactivity in the body (Payne et al., 2015). Then – a little at a time – the patient can turn their attention again to the disturbance and slowly regain ANS balance. For Molly this intervention ostensibly created the physiologic equivalent of emotional “space” from which she could more deliberately address her experience in the present moment. She can consider alternatives to her prior beliefs of danger in the world, even ultimately allowing the open expression of vulnerability, with tears.

## Conclusion

Our patients come to therapy when habitual responses, which are embedded within their physiology, fail to produce expected or desired outcomes. Predictive processing theories of the brain as an inference machine cast valuable light on how such dysfunctional patterns of responding can come about in infancy and be highly resistant to change. In order to change a prior it is necessary to act on the interoceptive system that created that prior in the first place.

Increasing attention to interoceptive sensation changes the balance of precision between the current interoceptive sensation and the “stubborn prior.” This change in precision can update a resistant prior and in doing so increase the patient’s ability to “mentalize interoception,” allowing alternative hypotheses to be generated about subjective experience. Intervening to influence precision similarly supports the patient’s efforts to bring emotion into awareness, which increases opportunities for their verbal expression – an important outcome of any therapeutic encounter.

We propose that the crucial point of access, within the therapeutic relationship is for the patient to focus attention onto their current internal bodily sensations (their interoception). Attention to the body, and the feelings that accompany this, sets in train a series of responses that may permit updating of default/habitual beliefs and the expectations that cause the patient distress in their current relationship to themselves, others and the world. We describe how this can re-calibrate the patient’s interoceptive responses, increase emotional awareness, strengthen evaluative thought patterns and allow the patient the flexibility to discern what is real and present in any given moment.

## Author Contributions

PD initiated the manuscript. Both authors contributed equally to the composition of the manuscript.

## Conflict of Interest

The authors declare that the research was conducted in the absence of any commercial or financial relationships that could be construed as a potential conflict of interest.

## Footnotes

1 Researchers in neuroscience have studied interoception from a variety of vantage points which has resulted in a range of definitions and perspectives. Discussion of different facets of interoception are beyond the scope of this paper but see Ceunen et al. (2016), Khalsa and Lapidus (2016), and Duquette (2017) for overviews of how the study of interoception has changed over the last century.

2 Predictive processing describes how this inferential activity may be organized within the hierarchical systems in the brain to produce generative models that are then used to infer (to generate) the causes of incoming sensory data (i.e., to explain it), whatever the form that data takes – whether interoceptive or exteroceptive Friston (2010).

3 Frith (2007) neatly describes Bayes’ Theorem as: “*Given some phenomenon (A) that we want to know about, and an observation (X) that is evidence relating to A, Bayes’ Theorem tells how much we should update our knowledge of A, given the new evidence X.*”
